# Assessment of Preoperative Multivitamin Use on the Impact on Micronutrient Deficiencies in Patients with Obesity Prior to Metabolic Bariatric Surgery

**DOI:** 10.1007/s11695-025-07853-1

**Published:** 2025-04-08

**Authors:** Johannes Sander, Bart Torensma, Jacqueline Siepe, Torsten Schorp, Thilo Schulte, Christine Schmeer, Hannes Gögele, Inga Böckelmann, Andrea Grabenhorst, Ildiko Ockert-Belz, Frits Berends, Edo Aarts

**Affiliations:** 1 Schön Klinik Hamburg Eilbek, Hamburg, Germany; 2 Erasmus MC, Rotterdam, The Netherlands; 3 WeightWorks Clinics, Amersfoort, The Netherlands

**Keywords:** Metabolic bariatric surgery, Micronutrient deficiencies, Multivitamin supplementation, Preoperative care, Obesity

## Abstract

**Background:**

Most patients achieve successful weight loss following metabolic bariatric surgery (MBS), but they face an increased risk of micronutrient deficiencies due to altered gastrointestinal physiology and dietary restrictions. This study evaluated the impact of a specialized multivitamin on blood serum levels before MBS.

**Methods:**

A prospective, within-patient comparison trial was conducted between January and July 2023 at a large bariatric clinic. Differences in serum micronutrient levels between baseline and the 3-month follow-up were assessed, along with changes in the prevalence of micronutrient deficiencies.

**Results:**

Of 120 patients recruited, 99 (82.5%) completed the 3-month follow-up. Significant changes were observed in 13 of 21 serum parameters (61.9%). Ten parameters, including iron, vitamin K_1_, zinc, C-reactive protein, hemoglobin, hematocrit, mean corpuscular volume, calcium, folic acid, and vitamin D, showed significant increases (*p* < 0.001). Conversely, magnesium, phosphate, and albumin levels significantly decreased (*p* < 0.001). Among 21 parameters, deficiencies were identified in 17 (80.1%), with prevalence rates ranging from 1.0% (copper) to 88.8% (vitamin D). After 3 months, significant reductions in deficiency prevalence were observed for iron, folic acid, and vitamin D. However, phosphate deficiency increased significantly, from 2.1 to 19.8% (*p* < 0.001).

**Conclusions:**

Micronutrient deficiencies are prevalent in patients with obesity. Preoperative specialized multivitamin supplementation effectively reduces key deficiencies, particularly in iron, folic acid, and vitamin D. Future research should address residual deficiencies and evaluate long-term outcomes of prolonged supplementation.

**Supplementary Information:**

The online version contains supplementary material available at 10.1007/s11695-025-07853-1.

## Introduction

Obesity is a significant contributor to a broad spectrum of metabolic disorders, including type 2 diabetes, cardiovascular disease, dyslipidemia, hypertension, and certain cancers [1, 2]. Additionally, it is associated with various psychological challenges in specific individuals [3].

Given the inconclusive evidence regarding the long-term efficacy of medical treatments and lifestyle modifications, metabolic bariatric surgery (MBS) had been proposed as an effective intervention to achieve sustained weight loss in patients with severe obesity [4–6]. While most patients achieved successful weight loss following MBS, they faced an increased risk of micronutrient deficiencies due to altered gastrointestinal physiology and dietary restrictions [7–9]. Nutritional guidelines for MBS patients emphasized the long-term use of specialized postoperative multivitamins to prevent these deficiencies [10–12].

Extensive research had demonstrated the safety and efficacy of multivitamins, tailored specifically to meet the nutritional needs of bariatric patients [13–18].

In addition to an increased risk for developing deficiencies after surgery, micronutrient deficiencies were already highly prevalent in patients with obesity compared to those with a healthy weight [9, 19–24]. Several factors contribute to this increased prevalence, including limited dietary diversity [25], dilution effects caused by increased serum volume, fat and muscle mass, and larger liver size; and obesity-related comorbidities such as liver steatosis [26], diabetes [27], inflammation [28, 29], and hormonal dysregulation [30, 31].

Moreover, inadequate intake or absorption of specific vitamins and minerals could disrupt the intricate balance between micronutrients, potentially compounding deficiencies. Micronutrients interact in complex ways, where a deficiency in one may affect the absorption or metabolism of others, potentially compounding nutritional challenges. For instance, low vitamin D levels can impair calcium absorption, increasing the risk of bone disorders. Consequently, nutritional guidelines emphasized the importance of comprehensive nutritional assessments before bariatric surgery, including preoperative screening for deficiencies and other health parameters [10–12].

Preoperative assessments includes screening for micronutrient deficiencies, diabetes, dyslipidemia, and renal function, with identified deficiencies corrected as clinically indicated before surgery.

Preoperative nutritional optimization is crucial for patients undergoing MBS. Major societies, including the American Society of Metabolic and Bariatric Surgery, the British Obesity & Metabolic Surgery Society, and the Canadian Adult Obesity Clinical Practice Guidelines, provide comprehensive preoperative micronutrient screening and supplementation recommendations [11, 12, 32].

Clinical studies have evaluated the safety and efficacy of post-bariatric specialized multivitamins tailored to the nutritional needs of various types of surgeries [7, 33, 34].

To our knowledge, there are no studies on the effect of specialized multivitamin supplementation on correcting blood serum values before MBS.

Our study aimed to evaluate the effectiveness of a comprehensive supplementation protocol in addressing preoperative micronutrient deficiencies.

## Methods

### Study Design

This was a prospective, within-patient comparison trial conducted between January 2023 and July 2023 at a large bariatric clinic. Patients met the International Federation for the Surgery of Obesity and Metabolic Disorders (IFSO) criteria after multidisciplinary screening and consented to undergo primary metabolic bariatric surgery. The study received approval from the institutional ethics committee (Ethical Board Registration Number: 2022-100796-BO-ff) and complied with the ethical standards of the 1964 Declaration of Helsinki. Written informed consent was obtained from all participants before their inclusion in the study.

### Study Objectives

All patients received a specialized multivitamin developed for pre-operative intake (WLS Start) for 3 months in addition to standard care, which included nutritional, physical, and psychological therapy.

The*primary objective* was to determine the within-patient differences in serum blood levels of micronutrients between baseline and the 3-month follow-up.

The *secondary objective* was to evaluate the percentage change in the prevalence of micronutrient deficiencies within patients between baseline and 3 months.

### Inclusion and Exclusion Criteria

Patients aged 18 to 65 years with a body mass index (BMI) ≥ 40 kg/m^2^, or a BMI ≥ 35 kg/m^2^ with associated medical problems such as type 2 diabetes mellitus (T2DM), hypertension, obstructive sleep apnea syndrome (OSAS), or hypercholesterolemia, were included in the study. Patients with a history of gastrointestinal diseases, such as celiac disease or inflammatory bowel disease, addiction behavior, suspected compliance issues, renal or hepatic insufficiency, or those unable to swallow the supplement, were excluded.

### Data Collection

This data collection process provided a comprehensive assessment of the impact of WLS Start supplementation. All the patients were required to discontinue over-the-counter (OTC) supplementation.

Data were collected at baseline (after MBS approval but before supplementation) and 3 months post-supplementation initiation. Information collected included demographic characteristics such as gender, age, and BMI; associated medical problems, including T2DM, hypertension, OSAS, and hypercholesterolemia; baseline use of vitamins and supplements; and any adverse effects or complaints related to supplement use.

### Laboratory Assessment

Peripheral blood samples were collected and allowed to clot at room temperature for 30 min. The samples were then centrifuged at 4000 rpm for 10 min at 4 °C, and the resulting serum was aliquoted and stored at − 80 °C until analysis. All laboratory analyses were conducted following standardized operating procedures to ensure accuracy and reproducibility.

The parameters with deficiencies based on the specified reference ranges are in Appendix 1. These parameters were selected to evaluate the nutritional and biochemical status of the participants comprehensively. Any values falling outside the specified reference ranges were classified as deficiencies/hypervitaminoses and further analyzed in the context of the study.

### Composition of the Supplement

WLS Start (FitForMe, the Netherlands) was administered once daily. The supplement contains the following micronutrients and dosages, with the respective percentage of the recommended dietary allowance (full description in Appendix 2).

This formulation is based on an elaborate literature review specifically designed to address the unique nutritional needs of patients preparing for MBS. It provided higher concentrations of essential micronutrients, such as vitamin B_12_, vitamin D, iron, and zinc, to mitigate the risk of deficiencies common in patients with obesity. The supplement was manufactured and administered following regulatory standards to ensure consistency and safety.

### Statistical Analysis

Descriptive and inferential statistical methods were applied. The normality of the data was assessed using the Kolmogorov–Smirnov test, Q–Q plots, and Levene’s test for equality of variances. Categorical variables were presented as counts and percentages (*n*, %). At the same time, continuous data were expressed as mean ± standard deviation (SD) for normally distributed variables and as median with interquartile range for skewed distributions.

Within-patient comparisons of categorical variables were analyzed using the McNemar test. For continuous data, independent-samples test and paired Student’s *t*-test were employed for normally distributed variables, while the Wilcoxon signed-rank test was used for non-normally distributed variables. Univariate and multivariable logistic regression analyses were performed using a backward selection method to identify and adjust for potential confounding factors. Statistical significance was defined as a *p*-value ≤ 0.05. All statistical analyses were conducted using R software version 4.1.3.

### Sample Size

The sample size was calculated using OpenEpi.com based on a clinically relevant reduction in ferritin deficiencies of 50%, assuming a baseline deficiency prevalence of 14% (SD: 15%), a 95% confidence interval (CI), and 80% power. The required sample size was determined to be 73 patients. Accounting for an anticipated drop-out rate at 3 months primarily due to non-adherence to multivitamin supplementation, the total sample size for the study was set at 120 patients.

## Results

### Baseline Characteristics

A total of 120 patients were recruited for the study, of whom 99 (82.5%) completed the 3-month follow-up period. The majority of participants were female (60.8%), with a mean age of 42.3 ± 11.81 years. The mean BMI at baseline was 47.4 ± 7.1 kg/m^2^.

Participants presented with various associated medical problems. The prevalence of hypertension was 49.2%, dyslipidemia 57.5%, T2DM 21.7%, hypothyroidism 23.3%, asthma 15.0%, depression 52.5%, gastroesophageal reflux disease 49.2%, and OSAS 32.5%. No participants had a diagnosis of coronary artery disease.

### Preoperative Supplement Use

At baseline, 19.9% of participants reported using OTC multivitamins or individual supplements. Furthermore, 11.6% used vitamin D, 2.5% vitamin B, 0.8% vitamin C, 1.6% vitamin K, 5.8% zinc, 3.3% magnesium, and 2.5% selenium.

### Changes in Blood Levels

Across the 21 measured parameters, no cases of hypervitaminosis were observed during the study. Thirteen parameters (61.9%) exhibited statistically significant changes in serum levels between baseline and follow-up. Nine parameters (42.8%) showed a significant increase in mean values, including iron, vitamin K_1_, zinc, C-reactive protein (CRP), hemoglobin, hematocrit, mean corpuscular volume (MCV), calcium, folic acid, and vitamin D. Conversely, three parameters (13.6%) displayed significant minimal decreases in mean values with a mean difference (95% CI) for magnesium of − 0.01 (− 0.03; 0.00), phosphate − 1.12 (− 0.17; − 0.07), and albumin − 0.89 (− 1.42; − 0.35) (*p* ≤ 0.001).

The three parameters with the most significant mean changes were iron (2.12; 95% CI: 1.12–3.12, *p* < 0.001), folic acid (10.92; 95% CI: 9.54–12.29, *p* < 0.001), and vitamin D (11.07; 95% CI: 9.38–12.76, *p* < 0.001) (Table 1).

**Table 1:** Change in blood level before and after 3 months of use of the supplement

Variable	*N*	Mean change	95% confidence interval	*p*-value
Iron	95	2.12	[1.12; 3.12]	< 0.001
Magnesium	95	− 0.01	[− 0.03; 0.00]	0.023
Vitamin A	99	0.01	[− 0.03; 0.05]	0.579
Vitamin E	99	0.15	[− 0.68; 0.97]	0.726
Vitamin K_1_	98	0.10	[− 0.01; 0.22]	0.073
Vitamin K_2_ (MK4)	98	− 0.03	[− 0.14; 0.07]	0.528
Vitamin K_2_ (MK7)	98	0.01	[− 0.08; 0.11]	0.747
Copper	99	− 0.09	[− 0.61; 0.43]	0.744
Zinc	92	61.19	[21.98; 100.41]	0.003
CRP	59	2.16	[0.02; 4.30]	0.048
Hemoglobin	99	0.37	[0.11; 0.63]	0.005
Hematocrit	99	0.01	[0.01; 0.02]	< 0.001
MCV	99	1.06	[0.54; 1.59]	< 0.001
Calcium	95	0.06	[0.04; 0.09]	< 0.001
Phosphate	96	− 0.12	[− 0.17; − 0.07]	< 0.001
Albumin	96	− 0.89	[− 1.42; − 0.35]	0.001
Ferritin	96	− 8.16	[− 17.10; 0.77]	0.073
Folic acid	94	10.92	[9.54; 12.29]	< 0.001
Vitamin B_12_	95	− 13.40	[− 45.56; 18.76]	0.410
Vitamin D	80	11.07	[9.38; 12.76]	< 0.001
Parathyroid hormone	96	− 6.85	[− 12.02; − 1.68]	0.010

*MCV* mean corpuscular volume, *CRP*C-reactive protein

### Deficiencies

Among the 21 assessed parameters, deficiencies were identified in 16 (76.2%) parameters, with prevalence rates ranging from 1.0% (copper) to 88.8% (vitamin D) of the patients. No deficiencies were observed in vitamin K_2_ (MK4), vitamin B_12_, or parathyroid hormone.

At the 3-month follow-up, deficiencies were identified in 15 assessed parameters, with prevalence rates ranging from 1 to 50% of patients.

After 3 months, 3 out of 21 micronutrients demonstrated a significant reduction in deficiency prevalence between baseline and follow-up. *Iron* deficiency decreased from 30.5 to 15.8% (*p* = 0.003). *Folic acid* deficiency showed a substantial decline, from 41.5 to 7.4% (*p* ≤ 0.001), and *vitamin D* deficiency improved significantly from 88.8 to 50.0% (*p* ≤ 0.001). However, *phosphate* deficiency increased significantly, rising from 2.1% at baseline to 19.8% at follow-up (*p* ≤ 0.001) (from 2 to 19 patients) (Table 2).

**Table 2:** Change of deficiencies

Variable	Proportion baseline	Proportion follow-up	*p*-value
Iron	29/95 (30.5%)	15/95 (15.8%)	0.003
Magnesium	3/95 (3.2%)	3/95 (3.2%)	1.000
Vitamin A	2/99 (2.0%)	5/99 (5.1%)	0.375
Vitamin E	1/99 (1.0%)	0/99 (0%)	1.000
Vitamin K_1_	3/98 (3.1%)	7/98 (7.1%)	0.219
Vitamin K_2_ (MK4)	0/98 (0%)	0/98 (0%)	n.a
Vitamin K_2_ (MK7)	3/98 (3.1%)	8/98 (8.2%)	0.180
Copper	1/99 (1.0%)	1/99 (1.0%)	1.000
Zinc	2/92 (2.2%)	4/92 (4.3%)	0.688
CRP	25/59 (42.4%)	19/59 (32.2%)	0.146
Hemoglobin	3/99 (3.0%)	0/99 (0%)	0.250
Hematocrit	2/99 (2.0%)	0/99 (0%)	0.500
MCV	16/99 (16.2%)	11/99 (11.1%)	0.125
Calcium	5/95 (5.3%)	1/95 (1.1%)	0.125
Phosphate	2/96 (2.1%)	19/96 (19.8%)	< 0.001
Albumin	15/96 (15.6%)	22/96 (22.9%)	0.039
Ferritin	2/96 (2.0%)	0/96 (0%)	0.500
Folic acid	39/94 (41.5%)	7/94 (7.4%)	< 0.001
Vitamin B_12_	0/95 (0%)	1/95 (1.1%)	1.000
Vitamin D	71/80 (88.8%)	40/80 (50.0%)	< 0.001
Parathyroid hormone	0/96 (0%)	1/96 (1.0%)	1.000

*MCV* mean corpuscular volume, *CRP*C-reactive protein, *n.a.* not available

### Correcting for Confounding Factor

When correcting for sex and BMI, significant differences emerged in the patterns of serum changes and deficiencies. For male participants with a BMI < 50, 23.8% of parameters, including calcium, phosphate, albumin, folic acid, and vitamin D, showed significant changes (*p* < 0.001). In males with BMI > 50, only three parameters (13.6%) showed significant increases, zinc, folic acid, and vitamin D, with zinc not being significant in the lower BMI group (Table 3).

**Table 3:** Correcting for confounding factors BMI < 50 and male gender, and correcting for confounding factors BMI > 50 and male gender

Variable	Mean change	95% confidence interval	*p*-value
Correcting for confounding factors BMI < 50 and male gender*			
Iron	1.27	[− 0.39; 2.94]	0.128
Magnesium	− 0.01	[− 0.03; 0.01]	0.261
Vitamin A	− 0.02	[− 0.10; 0.06]	0.671
Vitamin E	− 1.32	[− 2.73; 0.09]	0.066
Vitamin K_1_	0.03	[− 0.16; 0.21]	0.770
Vitamin K_2_ (MK4)	− 0.12	[− 0.36; 0.13]	0.341
Vitamin K_2_ (MK7)	− 0.09	[− 0.23; 0.04]	0.153
Copper	0.17	[− 0.79; 1.14]	0.714
Zinc	41.04	[− 42.89;124.97]	0.324
CRP	2.91	[− 0.29; 6.11]	0.071
Hemoglobin	0.17	[− 0.08; 0.42]	0.169
Hematocrit	0.00	[− 0.00; 0.01]	0.240
MCV	0.44	[− 0.44; 1.33]	0.312
Calcium	0.06	[0.03; 0.09]	< 0.001
Phosphate	− 0.18	[− 0.28;** − **0.08]	0.001
Albumin	− 1.63	[− 2.42;** − **0.85]	< 0.001
Ferritin	− 4.54	[− 27.07;18.00]	0.682
Folic acid	10.24	[7.67;12.82]	< 0.001
Vitamin B_12_	− 37.40	[− 93.48;18.68]	0.181
Vitamin D	11.04	[7.76;14.33]	< 0.001
Parathyroid hormone	− 4.43	[− 13.48; 4.61]	0.323
Correcting for confounding factors BMI > 50 and male gender*			
Iron	2.01	[− 0.05; 4.07]	0.054
Magnesium	− 0.03	[− 0.07; 0.01]	0.183
Vitamin A	0.02	[− 0.11; 0.15]	0.760
Vitamin E	0.81	[− 1.36; 2.98]	0.420
Vitamin K_1_	0.17	[− 0.04; 0.38]	0.102
Vitamin K_2_ (MK4)	− 0.09	[− 0.30; 0.13]	0.375
Vitamin K_2_ (MK7)	0.04	[− 0.13; 0.20]	0.613
Copper	0.12	[− 0.65; 0.89]	0.733
Zinc	149.20	[47.84;250.56]	0.009
CRP	1.97	[− 0.39; 4.34]	0.088
Hemoglobin	0.21	[− 0.22; 0.64]	0.296
Hematocrit	− 0.00	[− 0.03; 0.02]	0.605
MCV	0.30	[− 1.21; 1.81]	0.664
Calcium	0.05	[− 0.03; 0.12]	0.188
Phosphate	− 0.13	[− 0.28; 0.02]	0.083
Albumin	− 0.74	[− 2.49; 1.01]	0.363
Ferritin	− 26.80	[− 67.42;13.82]	0.170
Folic acid	11.04	[6.74;15.34]	< 0.001
Vitamin B_12_	24.20	[− 55.37;103.77]	0.509
Vitamin D	14.30	[8.42;20.18]	< 0.001
Parathyroid hormone	− 2.34	[− 21.52;16.84]	0.789

*MCV* mean corpuscular volume, *CRP* C-reactive protein

*Other potential confounding variables, including BMI as a continuous variable, did not significantly alter the observed outcomes in terms of changes in deficiencies

Among female participants with a BMI < 50, 28.5% of parameters showed significant changes, including hematocrit, MCV, phosphate, folic acid, vitamin B_12_, and vitamin D (*p* < 0.001). In contrast, women with a BMI > 50 exhibited more widespread changes, with 38.1% of parameters showing significant differences, including iron, CRP, hemoglobin, hematocrit, MCV, calcium, folic acid, vitamin D, and parathyroid hormone (*p* < 0.001) (Table 4).

**Table 4:** Correcting for confounding factors BMI < 50 and female gender, and correcting for confounding factors BMI > 50 and female gender

Variable	Mean change	95% confidence interval	*p*-value
Correcting for confounding factors BMI < 50 and female gender*			
Iron	1.68	[− 0.06; 3.43]	0.058
Magnesium	− 0.01	[− 0.03; 0.01]	0.382
Vitamin A	0.03	[− 0.02; 0.09]	0.234
Vitamin E	0.35	[− 0.78; 1.49]	0.533
Vitamin K_1_	0.14	[− 0.06; 0.34]	0.164
Vitamin K_2_ (MK4)	0.06	[− 0.11; 0.23]	0.454
Vitamin K_2_ (MK7)	− 0.03	[− 0.09; 0.03]	0.297
Copper	− 0.01	[− 0.71; 0.69]	0.976
Zinc	34.03	[− 15.60;83.66]	0.173
CRP	0.30	[− 3.58; 4.17]	0.876
Hemoglobin	0.72	[0.16; 1.28]	0.013
Hematocrit	0.02	[0.01; 0.02]	< 0.001
MCV	2.32	[1.45; 3.18]	< 0.001
Calcium	0.06	[0.02; 0.10]	0.003
Phosphate	− 0.15	[− 0.22;** − **0.08]	< 0.001
Albumin	− 0.76	[− 1.70; 0.17]	0.106
Ferritin	− 6.11	[− 15.60; 3.38]	0.200
Folic acid	10.92	[8.62;13.22]	< 0.001
Vitamin B_12_	− 30.19	[− 72.09;11.71]	0.153
Vitamin D	7.26	[4.06;10.46]	< 0.001
Parathyroid hormone	− 5.79	[− 12.95; 1.37]	0.110
Correcting for confounding factors BMI > 50 and female gender*			
Iron	3.31	[1.37; 5.25]	0.002
Magnesium	− 0.01	[− 0.04; 0.01]	0.305
Vitamin A	0.02	[− 0.03; 0.07]	0.447
Vitamin E	1.07	[− 0.92; 3.06]	0.278
Vitamin K_1_	0.12	[− 0.10; 0.33]	0.274
Vitamin K_2_ (MK4)	0.02	[− 0.12; 0.17]	0.774
Vitamin K_2_ (MK7)	0.14	[− 0.16; 0.44]	0.353
Copper	− 0.52	[− 1.80; 0.76]	0.408
Zinc	40.62	[− 42.83;124.06]	0.322
CRP	3.24	[1.49; 4.99]	0.002
Hemoglobin	0.46	[0.16; 0.76]	0.004
Hematocrit	0.02	[0.01; 0.03]	< 0.001
MCV	1.38	[0.42; 2.33]	0.007
Calcium	0.08	[0.05; 0.12]	< 0.001
Phosphate	− 0.03	[− 0.13; 0.06]	0.515
Albumin	− 0.31	[− 1.24; 0.62]	0.497
Ferritin	− 1.09	[− 11.05; 8.88]	0.823
Folic acid	11.51	[9.23;13.80]	< 0.001
Vitamin B_12_	− 3.09	[− 84.87;78.70]	0.938
Vitamin D	11.50	[9.31;13.69]	< 0.001
Parathyroid hormone	− 12.85	[− 24.02;** − **1.69]	0.026

*MCV* mean corpuscular volume, *CRP* C-reactive protein

*Other potential confounding variables, including BMI as a continuous variable, did not significantly alter the observed outcomes in terms of changes in deficiencies

Regarding deficiencies, iron had the most pronounced effect among women with a BMI > 50, decreasing from 43.5 to 17.4% (*p* < 0.001). The increase in phosphate deficiency was significant only among women with a BMI < 50, rising from 0.0% (*n* = 0) to 16.2% (*n* = 6) (*p* = 0.031). No such trend was observed in groups with higher BMI categories.

None of the other potential confounding variables, such as BMI treated as a continuous variable or the use of preoperativeOTC supplements, significantly influenced the observed changes or prevalence of deficiencies.

### Nausea and Side Effect of Supplement Use

There was no significant association between supplement use and nausea (*p* = 0.377). The majority of participants (67.7%) reported no nausea. Mild nausea was noted in 15 participants (15.2%), while 17.2% (17 participants) experienced nausea without vomiting.

During the supplementation period, 11.1% of participants reported mild side effects, including headache, bloating, flatulence, or an unpleasant taste. These effects were transient and did not necessitate discontinuation of the supplement. However, as dietary patterns and other lifestyle factors were not monitored, it remains unclear whether these side effects were exclusively attributable to the supplement**.**

## Discussion

This prospective study assessed the impact of specialized preoperative supplementation in patients with severe obesity undergoing MBS, focusing on identifying micronutrient deficiencies and evaluating the effectiveness of supplementation in addressing these deficiencies.

The deficiencies were identified in 76.2%, with statistically significant changes observed in 71.4% between baseline and follow-up.

Research on the use of specialized preoperative multivitamins in MBS patients are not known. A systematic review by Gudzune et al., which included 21,345 eligible patients, reported that presurgical testing for micronutrient deficiencies was performed in fewer than 25% of cases. Over a 36-month follow-up, the prevalence of micronutrient deficiencies increased across all MBS types, ranging from 20 to 88% [35].

More recently, a systematic review by Tang et al. examined 27 studies addressing micronutrient repletion before MBS, assessing micronutrient status both pre- and post-MBS. The interventions varied widely, including chronic oral supplementation with multivitamins (*n* = 21), megadoses of oral supplements (*n* = 1), intramuscular injections (*n* = 1), intravenous infusions (*n* = 1), and combined oral and injectable approaches (*n* = 3). The authors concluded that proactive micronutrient repletion schedules before MBS appear safe and feasible but noted variability in efficacy depending on the specific micronutrient, dose, duration, and delivery method (e.g., oral vs. infusion) [33].

Our results reported using OTC supplements prior to this study. However, this subgroup did not significantly reduce the prevalence of measured deficiencies or had a confounding effect on the changed micronutrient.

The study group’s active use of specialized supplementation (WLS Start) significantly reduced the prevalence of deficiency in 3 out of 22 micronutrients between baseline and the 3-month follow-up. However, deficiencies in 16 micronutrients persisted, potentially placing patients at a disadvantage when undergoing surgery. With 3 months, the study period was relatively short. Possibly, with longer use of preoperative supplementation, better blood serum values could have been reported. Since there were no hypervitaminosis, there is an opportunity for prolonged use.

Phosphate levels showed a mean decrease of − 0.12, accompanied by a significant increase in deficiency prevalence, rising from 3.8 to 23.1%. The observed increase in phosphate deficiencies in our study may potentially be linked to the use of ascorbic acid supplementation over the 3-month period. Ascorbic acid, particularly in higher doses, can contribute to metabolic acidosis, which in turn may initially result in transiently elevated serum phosphate levels. However, the resulting acidosis can lead to a compensatory increase in renal phosphate excretion over time, ultimately reducing serum phosphate concentrations [36]. This mechanism aligns with our findings of decreased phosphate levels and increased deficiency prevalence, but further research is needed to confirm this hypothesis and evaluate the extent to which ascorbic acid supplementation may contribute to phosphate imbalances in this population.

### Impact of Fewer Deficiencies in Vitamin D, Iron, and Folic Acid

Reducing deficiencies in vitamin D, iron, and folic acid is crucial for MBS patients due to their essential roles in metabolic and physiological processes.

Vitamin D deficiency, common in severe obesity and often exacerbated after MBS, plays a critical role in calcium absorption, bone health, immune function, and in reducing systemic inflammation. A recent systematic review by Giustina et al. emphasized the importance of assessing vitamin D levels both preoperatively and postoperatively in all patients undergoing MBS. The review further recommends high-dose vitamin D supplementation pre- and postoperatively, irrespective of the surgical procedure type, to address these deficiencies effectively [37].

Addressing this deficiency preoperatively minimizes the risks of postoperative complications such as bone demineralization and poor wound healing.

Iron deficiency, linked to anemia and fatigue, is often exacerbated by reduced intake and malabsorption after surgery. Adequate iron levels support hemoglobin synthesis and oxygen transport, which are essential for healing. Similarly, folic acid is vital for DNA synthesis, red blood cell production, and tissue repair, reducing risks of megaloblastic anemia and supporting recovery and support rapid cell turnover required during tissue repair after surgery.

In our study, iron had the most pronounced effect among women with a BMI > 50, decreasing from 43.5 to 17.4%. While the relationship between obesity and endometrial cancer is widely recognized, there is limited knowledge regarding the frequency of heavy menstrual bleeding among obese adolescents and adult women [38, 39].

### Rationale for Preoperative Supplementation

Many deficiencies are already present preoperatively due to limited dietary diversity, dilution effects from increased serum volume, expanded fat and muscle mass, and conditions like liver steatosis, diabetes, and chronic inflammation [9, 25–31]. If unaddressed, these deficiencies are likely to worsen postoperatively due to reduced nutrient intake and absorption.

Preoperative supplementation addressing existing deficiencies can potentially reduce risks during recovery, establish adherence to supplementation regimens, and optimize outcomes by supporting immune function wound healing, and may have a protective effect against hair loss with levels of zinc, folic acid, ferritin, and protein intake that are closely associated with the risk of hair loss, highlighting the importance of optimizing these factors before surgery, and overall metabolic health.

## Limitations

This study has several limitations that should be considered. A washout period was not applied, meaning that the effects of any prior supplementation or dietary habits could not be fully isolated from the intervention. Despite this, deficiencies were still present preoperatively in many participants, underscoring the need for tailored nutritional optimization prior to MBS.

Additionally, while the preoperative setting was particularly relevant for identifying and addressing deficiencies, the absence of a true control group limits the ability to attribute observed changes solely to the intervention. Potential confounding factors, such as differences in metabolism, associated medical problems, or baseline nutritional statuses, may also have influenced the results since we could not correct for all the factors due to low incidence.

The relatively short study duration of 3 months may not fully capture the long-term implications of preoperative supplementation, particularly for deficiencies that take longer to resolve or have extended effects on surgical outcomes.

Finally, the study’s focus on a specific preoperative bariatric population in a single healthcare setting may limit the generalizability of findings to other populations or healthcare systems.

These limitations highlight the need for future studies with robust designs, including randomized controlled trials, washout periods, longer follow-up durations, and more detailed monitoring of adherence and external supplement use, to validate these findings and better understand the long-term benefits of preoperative supplementation.

## Conclusion

This study highlights the prevalence of micronutrient deficiencies in patients with obesity and demonstrates the effectiveness of preoperative specialized multivitamin supplementation in reducing key deficiencies, including iron, folic acid, and vitamin D. Significant improvements in serum levels of critical nutrients such as zinc further underscore the benefits of tailored preoperative supplementation.

Future research is warranted to address residual deficiencies in other micronutrient and evaluate long-term outcomes associated with prolonged preoperative supplementation. Studies should also assess the effects after MBS and determine the burden of postoperative deficiencies after preoperative supplement use.

## Supplementary Information

Below is the link to the electronic supplementary material.

Supplementary file 1 (DOCX 15.3 KB)

Supplementary file 2 (DOCX 15.0 KB)

## Data Availability

Data is available with the corresponding author.

## References

[1] Guh DP, Zhang W, Bansback N, Amarsi Z, Birmingham CL, Anis AH. The incidence of co-morbidities related to obesity and overweight: a systematic review and meta-analysis. BMC Public Health. 2009;9:88. 19320986 10.1186/1471-2458-9-88 PMC2667420

[2] Khaodhiar L, McCowen KC, Blackburn GL. Obesity and its comorbid conditions. Clin Cornerstone. 1999;2:17–31. 10696282 10.1016/s1098-3597(99)90002-9

[3] Bremner J, Moazzami K, Wittbrodt M, et al. Diet, stress and mental health. Nutrients. 2020;12:2428. 32823562 10.3390/nu12082428 PMC7468813

[4] Cottam S, Cottam D, Cottam A, et al. The use of predictive markers for the development of a model to predict weight loss following vertical sleeve gastrectomy. Obes Surg. 2018;28:3769–74. 30039237 10.1007/s11695-018-3417-3

[5] Douketis JD, Macie C, Thabane L, Williamson DF. Systematic review of long-term weight loss studies in obese adults: clinical significance and applicability to clinical practice. Int J Obes. 2005;29:1153–67. 10.1038/sj.ijo.0802982 15997250

[6] Ikramuddin S, Korner J, Lee W-J, et al. Roux-en-Y gastric bypass vs intensive medical management for the control of type 2 diabetes, hypertension, and hyperlipidemia: the Diabetes Surgery Study randomized clinical trial. JAMA. 2013;309:2240. 23736733 10.1001/jama.2013.5835 PMC3954742

[7] Mohapatra S, Gangadharan K, Pitchumoni CS. Malnutrition in obesity before and after bariatric surgery. Dis Mon. 2020;66:100866. 31301800 10.1016/j.disamonth.2019.06.008

[8] Lupoli R, Lembo E, Saldalamacchia G, et al. Bariatric surgery and long-term nutritional issues. WJD. 2017;8:464. 29204255 10.4239/wjd.v8.i11.464 PMC5700383

[9] Van Rutte PWJ, Aarts EO, Smulders JF, et al. Nutrient deficiencies before and after sleeve gastrectomy. Obes Surg. 2014;24:1639–46. 24706197 10.1007/s11695-014-1225-y

[10] Mechanick JI, Apovian C, Brethauer S, et al. Clinical practice guidelines for the perioperative nutrition, metabolic, and nonsurgical support of patients undergoing bariatric procedures – 2019 update: cosponsored by American Association of Clinical Endocrinologists/American College of Endocrinology, The Obesity Society, American Society For Metabolic & Bariatric Surgery, Obesity Medicine Association, and American Society of Anesthesiologists. Endocr Pract. 2019;25:1–75. 10.4158/GL-2019-0406 31682518

[11] O’Kane M, Parretti HM, Pinkney J, et al. British Obesity and Metabolic Surgery Society Guidelines on perioperative and postoperative biochemical monitoring and micronutrient replacement for patients undergoing bariatric surgery—2020 update. Obes Rev. 2020;21:e13087. 32743907 10.1111/obr.13087 PMC7583474

[12] Parrott J, Frank L, Rabena R, et al. American Society for Metabolic and Bariatric Surgery Integrated Health Nutritional Guidelines for the Surgical Weight Loss Patient 2016 Update: Micronutrients. Surg Obes Relat Dis. 2017;13:727–41. 28392254 10.1016/j.soard.2016.12.018

[13] Dogan K, Aarts EO, Koehestanie P, et al. Optimization of vitamin suppletion after Roux-En-Y gastric bypass surgery can lower postoperative deficiencies: a randomized controlled trial. Medicine. 2014;93:e169. 25437032 10.1097/MD.0000000000000169 PMC4616370

[14] Heusschen L, Berendsen AAM, Cooiman MI, et al. Optimizing multivitamin supplementation for sleeve gastrectomy patients. Obes Surg. 2021;31:2520–8. 33624212 10.1007/s11695-021-05282-4 PMC8113195

[15] Heusschen L, Schijns W, Ploeger N, et al. The true story on deficiencies after sleeve gastrectomy: results of a double-blind RCT. Obes Surg. 2020;30:1280–90. 31776782 10.1007/s11695-019-04252-1

[16] Homan J, Schijns W, Aarts EO, et al. An optimized multivitamin supplement lowers the number of vitamin and mineral deficiencies three years after Roux-en-Y gastric bypass: a cohort study. Surg Obes Relat Dis. 2016;12:659–67. 26947791 10.1016/j.soard.2015.12.010

[17] Smelt HJM, Van Loon S, Pouwels S, et al. Do specialized bariatric multivitamins lower deficiencies after sleeve gastrectomy? Obes Surg. 2020;30:427–38. 31749110 10.1007/s11695-019-04191-x

[18] Schijns W, Schuurman LT, Melse-Boonstra A, et al. Do specialized bariatric multivitamins lower deficiencies after RYGB? Surg Obes Relat Dis. 2018;14:1005–12. 29903686 10.1016/j.soard.2018.03.029

[19] Al-Mutawa A, Al-Sabah S, Anderson AK, et al. Evaluation of nutritional status post laparoscopic sleeve gastrectomy—5-year outcomes. Obes Surg. 2018;28:1473–83. 29197046 10.1007/s11695-017-3041-7

[20] Ernst B, Thurnheer M, Schmid SM, et al. Evidence for the necessity to systematically assess micronutrient status prior to bariatric surgery. Obes Surg. 2009;19:66–73. 18491197 10.1007/s11695-008-9545-4

[21] Frame-Peterson LA, Megill RD, Carobrese S, et al. Nutrient deficiencies are common prior to bariatric surgery. Nut in Clin Prac. 2017;32:463–9. 10.1177/0884533617712701 28636832

[22] Krzizek E-C, Brix JM, Herz CT, et al. Prevalence of micronutrient deficiency in patients with morbid obesity before bariatric surgery. Obes Surg. 2018;28:643–8. 28849358 10.1007/s11695-017-2902-4

[23] Moizé V, Deulofeu R, Torres F, et al. Nutritional intake and prevalence of nutritional deficiencies prior to surgery in a Spanish morbidly obese population. Obes Surg. 2011;21:1382–8. 21298509 10.1007/s11695-011-0360-y

[24] Peterson LA, Cheskin LJ, Furtado M, et al. Malnutrition in bariatric surgery candidates: multiple micronutrient deficiencies prior to surgery. Obes Surg. 2016;26:833–8. 26297429 10.1007/s11695-015-1844-y

[25] Damms-Machado A, Weser G, Bischoff SC. Micronutrient deficiency in obese subjects undergoing low calorie diet. Nutr J. 2012;11:34. 22657586 10.1186/1475-2891-11-34 PMC3404899

[26] Targher G, Bertolini L, Scala L, et al. Associations between serum 25-hydroxyvitamin D3 concentrations and liver histology in patients with non-alcoholic fatty liver disease. Nutr Metab Cardiovasc Dis. 2007;17:517–24. 16928437 10.1016/j.numecd.2006.04.002

[27] Palomer X, González-Clemente JM, Blanco-Vaca F, et al. Role of vitamin D in the pathogenesis of type 2 diabetes mellitus. Diabetes Obes Metab. 2008;10:185–97. 18269634 10.1111/j.1463-1326.2007.00710.x

[28] Cepeda-Lopez AC, Aeberli I, Zimmermann MB. Does obesity increase risk for iron deficiency? A review of the literature and the potential mechanisms. Int J Vitam Nutr Res. 2010;80:263–70. 21462109 10.1024/0300-9831/a000033

[29] Ausk KJ, Ioannou GN. Is obesity associated with anemia of chronic disease? A population-based study. Obesity. 2008;16:2356–61. 18719644 10.1038/oby.2008.353

[30] Kahn SE, Hull RL, Utzschneider KM. Mechanisms linking obesity to insulin resistance and type 2 diabetes. Nature. 2006;444:840–6. 17167471 10.1038/nature05482

[31] Kumar R, Mal K, Razaq MK, et al. Association of leptin with obesity and insulin resistance. Cureus [Internet]. 2020. Available from: https://www.cureus.com/articles/47751-association-of-leptin-with-obesity-and-insulin-resistance. Accessed 16 Mar 2025. 10.7759/cureus.12178 PMC7815269 33489589

[32] Canadian Adult Obesity Clinical Practice Guidelines. 2025. Available from: https://obesitycanada.ca/healthcare-professionals/clinical-practice-guideline/. Accessed 16 Mar 2025.

[33] Tang X, Reidlinger DP, Crichton M, et al. Preoperative micronutrient repletion strategies in metabolic and bariatric surgery: a systematic review. J Acad Nutr Diet. 2024;S2212267224008645. 10.1016/j.jand.2024.09.007 39306086

[34] Kamal FA, Fernet LY, Rodriguez M, et al. Nutritional deficiencies before and after bariatric surgery in low- and high-income countries: prevention and treatment. Cureus [Internet]. 2024. Available from: https://www.cureus.com/articles/233173-nutritional-deficiencies-before-and-after-bariatric-surgery-in-low--and-high-income-countries-prevention-and-treatment. Accessed 16 Mar 2025. 10.7759/cureus.55062 PMC10977614 38550458

[35] Gudzune KA, Huizinga MM, Chang H-Y, et al. Screening and diagnosis of micronutrient deficiencies before and after bariatric surgery. Obes Surg. 2013;23:1581–9. 23515975 10.1007/s11695-013-0919-x PMC3740071

[36] Salcedo-Betancourt JD, Moe OW. The effects of acid on calcium and phosphate metabolism. IJMS. 2024;25:2081. 38396761 10.3390/ijms25042081 PMC10889523

[37] Giustina A, Di Filippo L, Facciorusso A, et al. Vitamin D status and supplementation before and after bariatric surgery: recommendations based on a systematic review and meta-analysis. Rev Endocr Metab Disord. 2023;24:1011–29. 37665480 10.1007/s11154-023-09831-3 PMC10698146

[38] McDonald ME, Bender DP. Endometrial cancer. Obstet Gynecol Clin North Am. 2019;46:89–105. 30683268 10.1016/j.ogc.2018.09.006

[39] Calle EE. Rodriguez C, Walker-Thurmond K, Thun MJ. Overweight, obesity, and mortality from cancer in a prospectively studied cohort of U.S. adults. N Engl J Med. 2003;348:1625–38. 10.1056/NEJMoa021423. 10.1056/NEJMoa021423 12711737

