# Correction to: Assessment of Preoperative Multivitamin Use on the Impact on Micronutrient Deficiencies in Patients with Obesity Prior to Metabolic Bariatric Surgery

**DOI:** 10.1007/s11695-025-07970-x

**Published:** 2025-06-11

**Authors:** Johannes Sander, Bart Torensma, Jacqueline Siepe, Torsten Schorp, Thilo Schulte, Christine Schmeer, Hannes Gögele, Inga Böckelmann, Andrea Grabenhorst, Ildiko Ockert-Belz, Frits Berends, Edo Aarts

**Affiliations:** 1 Schön Klinik Hamburg Eilbek, Hamburg, Germany; 2 Erasmus MC, Rotterdam, The Netherlands; 3 WeightWorks Clinics, Amersfoort, The Netherlands

**Correction to: Obesity Surgery (2025) 35:1818-1826**

10.1007/s11695-025-07853-1

During revision of the manuscript, the *n*% was changed and wrongly published.

The corrected text is as follows:

In the Abstract:

Among 21 parameters, deficiencies were identified in 18 (85.7%), with prevalence rates ranging from 1.0% (copper) to 88.8% (vitamin D).

In the main text:

Among the 21 assessed parameters, deficiencies were identified in 18 (85.7%), with prevalence rates ranging from 1.0% (copper) to 88.8% (vitamin D) of the patients.

In the Discussion section:

The deficiencies were identified in 85.7%, with statistically significant changes observed in 71.4% between baseline and follow-up.

