# Supplementary material for: Assessment of Preoperative Multivitamin Use on the Impact on Micronutrient Deficiencies in Patients with Obesity Prior to Metabolic Bariatric Surgery
Source: Obes Surg. 2025 Apr 8;35(5):1818–26. doi: 10.1007/s11695-025-07853-1 (PMC12065735; doi:10.1007/s11695-025-07853-1)
Supplement: Supplementary file 1 — (DOCX 15.3 KB) [file 11695_2025_7853_MOESM1_ESM.docx]

Appendix 1: Lab assessment for specified reference ranges

- **Hemoglobin:** 12–16 g/dL
- **Mean Corpuscular Volume (MCV):** 82–98 fL
- **Iron:** 9.0–30.4 µmol/L
- **Ferritin:** 10–291 ng/mL
- **Folic Acid:** 6.0–17.0 ng/mL
- **Vitamin B12:** 211-911 pg/ml
- **C-Reactive Protein (CRP):** <0.5 mg/L
- **Vitamin D (25-OH):** 25–130 µg/L
- **Parathyroid Hormone (PTH):** 15–65 pg/mL
- **Calcium:** 2.15–2.58 mmol/L
- **Albumin:** 35–53 g/L
- **Zinc:** 600–1200 µg/L
- **Phosphate:** 0.87–1.45 mmol/L
- **Magnesium:** 0,65-1,07 mmol/l
- **Vitamin A:** 0.3–0.7 mg/L
- **Vitamin E:** 5–18 mg/L
- **Vitamin K:** K1 0,13-1,19 µg/l

**K2** (MK-4) 0,1-0,86 µg/l

**K2** (MK-7) 0,1-0,82 µg/l

- **Copper:** 11-22 µmol/l
