# Supplementary material for: Assessment of Preoperative Multivitamin Use on the Impact on Micronutrient Deficiencies in Patients with Obesity Prior to Metabolic Bariatric Surgery
Source: Obes Surg. 2025 Apr 8;35(5):1818–26. doi: 10.1007/s11695-025-07853-1 (PMC12065735; doi:10.1007/s11695-025-07853-1)
Supplement: Supplementary file 2 — (DOCX 15.0 KB) [file 11695_2025_7853_MOESM2_ESM.docx]

**Appendix 2: Dosage of WLS Start**

| **Micronutrient** | **Dosage** | **Unit** | **RDA (%)** |
| --- | --- | --- | --- |
| Vitamin A | 1000 | mcg | 125 |
| Beta-carotene | 0.2 | mg | 469 |
| Vitamin B1 | 5.5 | mg | 500 |
| Vitamin B2 | 0.7 | mg | 50 |
| Vitamin B3 | 8 | mg | 50 |
| Vitamin B5 | 6 | mg | 100 |
| Vitamin B6 | 0.7 | mg | 50 |
| Vitamin B8 | 50 | mcg | 100 |
| Vitamin B9 | 600 | mcg | 300 |
| Vitamin B12 | 100 | mcg | 4000 |
| Vitamin C | 250 | mg | 313 |
| Vitamin D | 75 | mcg | 1500 |
| Vitamin E | 12 | mg | 100 |
| Iron | 24 | mg | 172 |
| Zinc | 22.5 | mg | 225 |
| Copper | 2.25 | mg | 225 |
| Chromium | 80 | mcg | 200 |
| Iodine | 150 | mcg | 100 |
| Selenium | 100 | mcg | 182 |
| Manganese | 2 | mg | 100 |
| Molybdenum | 50 | mcg | 100 |
| Magnesium | 16–24 | mg | — |
